# Time-Lagging Interplay Effect and Excess Risk of Meteorological/Mosquito Parameters and Petrochemical Gas Explosion on Dengue Incidence

**DOI:** 10.1038/srep35028

**Published:** 2016-10-13

**Authors:** Ko Chang, Chaur-Dong Chen, Chien-Ming Shih, Tzu-Chi Lee, Ming-Tsang Wu, Deng-Chyang Wu, Yen-Hsu Chen, Chih-Hsing Hung, Meng-Chieh Wu, Chun-Chi Huang, Chien-Hung Lee, Chi-Kung Ho

**Affiliations:** 1 Division of Infectious Diseases, Department of Internal Medicine, Kaohsiung Medical University Hospital, College of Medicine, Kaohsiung Medical University, Kaohsiung, Taiwan; 2 Department of Internal Medicine, Kaohsiung Municipal Hsiao-Kang Hospital, Kaohsiung Medical University, Kaohsiung, Taiwan; 3 Bureau of Public Health, Kaohsiung City Government, Kaohsiung, Taiwan; 4 Graduate Institute of Medicine, College of Medicine, Kaohsiung Medical University, Kaohsiung, Taiwan; 5 Research Center for Environmental Medicine, Kaohsiung Medical University, Kaohsiung, Taiwan; 6 Department of Public Health, College of Health Science, Kaohsiung Medical University, Kaohsiung, Taiwan; 7 Department of Community Medicine, Kaohsiung Medical University Hospital, Kaohsiung Medical University, Kaohsiung, Taiwan; 8 Department of Internal Medicine, Kaohsiung Municipal Ta-Tung Hospital, Kaohsiung Medical University, Kaohsiung, Taiwan; 9 Department of Pediatrics, Kaohsiung Municipal Hsiao-Kang Hospital, Kaohsiung Medical University, Kaohsiung, Taiwan; 10 Department of Laboratory Medicine, Kaohsiung Municipal Hsiao-Kang Hospital, Kaohsiung, Taiwan

## Abstract

In Kaohsiung, a metropolitan city in Taiwan at high risk of dengue epidemic, weather factors combined with an accidental petrochemical gas explosion (PGE) may affect mosquito‒human dynamics in 2014. Generalized estimating equations with lagged-time Poisson regression analyses were used to evaluate the effect of meteorological/mosquito parameters and PGE on dengue incidences (2000–2014) in Kaohsiung. Increased minimum temperatures rendered a 2- and 3-month lagging interactive effect on higher dengue risks, and higher rainfall exhibited a 1- and 2-month lagging interplay effect on lower risks (interaction, *P* ≤ 0.001). The dengue risk was significantly higher than that in a large-scale outbreak year (2002) from week 5 after PGE accident in 2014 (2.9‒8.3-fold for weeks 5‒22). The greatest cross-correlation of dengue incidences in the PGE-affected and PGE-neighboring districts was identified at weeks 1 after the PGE (*r*_s_ = 0.956, *P* < 0.001). Compared with the reference years, the combined effect of minimum temperature, rainfall, and PGE accounted for 75.1% of excess dengue risk in 2014. In conclusion, time-lagging interplay effects from minimum temperature and rainfall may be respectively associated with early and near environments facilitating dengue transmission. Events that interact with weather and influence mosquito‒human dynamics, such as PGEs, should not be ignored in dengue prevention and control.

Dengue, a mosquito-borne viral infection, circulates in all tropical and numerous subtropical areas worldwide through endemic and epidemic transmission modes^1^. Nearly 390 million dengue cases are reported annually worldwide, with 96 million cases exhibiting some level of dengue severity^1^. In 2012, a global study estimated that 3.97 billion people in 128 countries are at risk of dengue infection^2^. Dengvaxia (CYD-TDV) is the first vaccine licensed for preventing dengue; as of June 2016, it has been approved for use in Mexico, Brazil, El Salvador, the Philippines, and Costa Rica^3^. No therapeutic drugs or effective vector control programs have been implemented^1^,^4^.

Taiwan, situated in the western Pacific Ocean, has a warm tropical/subtropical and highly humid climate, suitable for the breeding and biting activity of dengue vector mosquitos (*Aedes aegypti* and *Aedes albopictus*). Dengue has been circulating in Southern Taiwan since the late 19th century, and Kaohsiung has been the major epidemic city in recent decades^5^,^6^. In 2000–2014, 81.7% of all newly diagnosed cases of indigenous dengue nationwide occurred in Kaohsiung^7^; the number of dengue cases in two large-scale epidemics in the city was 5,336 and 15,492 (90.2% and 96.8% of all national cases) in 2002 and 2014, respectively^8^. Fluctuations in temperature, humidity, precipitation, and mosquito populations alter the spatial and temporal ecological dynamics of the dengue virus^9^,^10^. However, the effects of meteorological and mosquito parameters on dengue incidence in Kaohsiung remain unclear.

Kaohsiung has long been a center of heavy industry in Taiwan; numerous pipelines have been constructed underneath city streets by petrochemical factories. On July 31, 2014, an underground gas explosion induced by a propylene leak occurred in the dengue-prevalent Kaohsiung districts of Qianzhen and Lingya, causing 32 deaths and 321 injuries (video report: http://edition.cnn.com/videos/world/2014/08/01/pkg-coren-taiwan-gas-explosions-wrap.cnn)^11^,^12^,^13^. The petrochemical gas explosion (PGE) destroyed street gutters, damaged buildings, and produced numerous potholes and open spaces^14^. This was followed by rainfall for several days and then by hot weather. These conditions created a physical environment conducive for habitats that facilitate the breeding and feeding activity of dengue vector mosquitos. In addition, vector control measures were interrupted temporarily, and the mobility of the residents living in and around the affected area increased after the PGE^15^. The climatic data indicated that the average temperature of Kaohsiung in 2014 was the highest of any year in the 2000–2014 study period. The PGE, along with the high temperature, induced great concern regarding whether and how such an accident, which may influence mosquito‒human dynamics, would affect dengue occurrence in a metropolitan city at high risk of dengue infection.

This study first evaluated the effect of meteorological and mosquito factors on dengue infection in Kaohsiung, and then investigated the individual and combined effects of meteorological/mosquito parameters and the PGE event on dengue incidence.

## Methods

### Study population

Kaohsiung, a city comprising 38 administrative areas in 2010, is a special municipality and has the second largest population in Taiwan (approximately 2.78 million). It is the industrial center of Southern Taiwan and is located at south of the Tropic of Cancer. Its climate is tropical, with monthly mean temperatures of 18 °C‒31 °C and a relative humidity of 63‒85%^16^. In this investigation, the study population was defined as people living in Kaohsiung city.

### Surveillance data

Dengue is a category II communicable disease, reporting of which within 24 h of diagnosis is legally mandatory in Taiwan. To monitor and control dengue efficiently, the Taiwan Centers for Disease Control (CDC) established a rapid reporting system for infectious diseases. This surveillance system for indigenous dengue cases uses notifications regarding suspected patients provided by physicians who diagnose dengue cases in clinics and hospitals. Imported dengue cases are monitored through fever screenings of incoming passengers at Taiwan entry ports. Only laboratory-confirmed dengue cases, verified using serological tests, are registered in the National Infectious Disease Statistics System (NIDSS)^7^.

In this study, dengue cases were defined as those serologically diagnosed with new indigenous dengue between January 2000 and December 2014. All weekly, monthly, and yearly patient data were obtained from the NIDSS^7^. Population data were collected from the Department of Household Registration, which systematically performs national population and housing censuses across Taiwan^17^. Because no weekly population data were available, monthly and weekly dengue incidence rates were calculated by dividing the number of monthly and weekly dengue cases by the corresponding mid-month population.

### Meteorological and mosquito parameters

The Taiwan Central Weather Bureau (CWB), which is responsible for government meteorological research and forecasting, has established 25 weather stations and 4 weather radar stations. To evaluate the association between meteorological factors and dengue incidence in the study population, detailed meteorological data, including daily maximum, average and minimum temperatures, rainfall intensity, and relative humidity from January 2000 to December 2014, were obtained from the CWB^16^. The monthly maximum and minimum temperatures were calculated by averaging the daily maximum and minimum temperatures, respectively, over a specified month. Monthly temperature range was determined using the difference between the minimum and maximum values of temperature. The Taiwan CDC measures four vector indices for monitoring mosquito density, including the Breteau index (BI), container index, house index, and adult index. Of these, BI is the most central and widely used one for monitoring dengue vector populations^18^. BI measures the number of positive containers for dengue vector mosquito larvae per 100 inspected houses. The percentage of days with a BI of >2 (ie, ≥10 containers having larvae per 100 inspected houses per day) in a month was used to estimate the potential risk of mosquito transmission. All BI data were obtained from the Department of Health of the Kaohsiung Government and from the CDC^7^,^19^.

### Statistical analysis

Generalized estimating equations (GEEs) with a lagged-time Poisson regression analysis were used to evaluate the effect of meteorological and mosquito factors on dengue incidence, and adjust for the correlation between dengue incidences within the same months^20^. Because any two dengue incidences within the same month may have a comparable correlation, the exchangeable correlation structure was employed for the GEE analysis. A complete multivariate Poisson regression model could be formulated as follows:


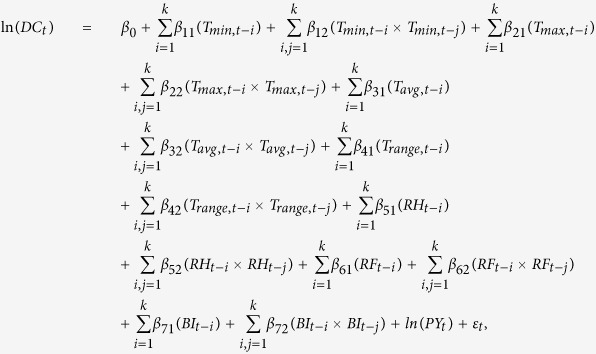


where *t* denotes the month of the observation; *DC*_*t*_ represents the dengue cases observed at month *t*; *T*_min_, *T*_max_, *T*_avg_, and *T*_range_ denote minimum, maximum, average, and range of monthly temperatures (°C), respectively; *RH*, *RF*, and *BI* indicate relative humidity (%), rainfall (100 mm), and the percentage of the days with BI >2 in a month, respectively; *i* and *j* denote the lag months; *k* represents the maximum lag, thus *t*−*i* and *t*−*j* in the subscript denote the analyzed month; β_0_ denotes the intercept; β_11–71_ and β_12–72_ represent the main and interaction effect coefficients of the covariates, respectively; and *PY*_*t*_ is an offset used to control for person-years at month *t*.

To investigate the univariate time-lagging effect on dengue incidence, single meteorological or mosquito time-lagging variables and their interaction terms were included in the GEE model. In the multivariate analysis, all environmental factors with time-lagging variables and their interaction terms were evaluated in the complete GEE model. Nonsignificant variables were removed through backward elimination and stepwise regression modeling. We used quasi-likelihood-based information criterion (QICu) to determine the most parsimonious regression model, in that a model with a smaller QICu value is preferred^21^. To facilitate the interpretation of interaction, all variables were centered before analysis by subtracting the corresponding mean from each observed value (distributions for the covariates are shown in Supplementary Information Table 1). Alternatively, we used BI data for 1‒6 months during 2000–2014 and GEE models with a linear link function to evaluate the association between the covariates obtained in the best-fitting model and dengue vector mosquito density index. In Taiwan, a drive to eliminate mosquito-breeding sites was promoted by the government and residents after a new wave of dengue infections occurred; these activities substantively affected natural BI values (the effect of BI on dengue incidence at 1-month lagging was negative: β = −0.017; Table 1). Because new waves of dengue infection regularly begin in July, BI values from January to June were considered more appropriate in response to natural situations.

Incidence rate ratios (IRRs) with corresponding 95% confidence intervals obtained from the GEE regression models were used to investigate the possible effects of PGE (which occurred at the 31st week in 2014) during various periods. The weekly dengue incidence rates in 2014 were compared with those in 2002 as well as 2006, 2010, and 2011 (2014 and 2002 were large-scale outbreak years, and 2006, 2010, and 2011 were moderate-scale outbreak years; annual dengue incidence distribution is shown in Supplementary Information Figure 1). The periods we investigated included 5‒30 and 1‒4 weeks before the PGE and 1‒4, 5‒8, 9‒12, 13‒16, 17‒20, and 21‒22 weeks after the PGE. The adjusted IRRs (aIRRs) for these periods were adjusted for all meteorological and mosquito parameters.

We next compared cross-time associations of weekly dengue incidences between 2002 (outbreak without PGE) and 2014 (outbreak with PGE): Spearman’s rank correlation coefficient (*r*_s_) with Bonferroni-adjusted *P* value was used for measuring the time cross-correlations between weekly dengue incidences in the PGE-affected districts Qianzhen and Lingya and subsequent dengue incidences in their neighboring districts Sanmin, Xiaogang, and Fengshan and in other Kaohsiung districts (33 districts in total).

GEE models with Poisson distribution were also applied to measure the effect of year and environmental parameters on dengue incidence. All years except 2002 and 2014 were considered the reference years (13 years). The degree to which meteorological and mosquito parameters explained the excess risk of dengue incidence in 2002 and 2014 compared with that in reference years was calculated as follows: (IRR_1_−IRR_2_)/(IRR_1_−1), where IRR_1_ denotes IRR for 2002 or 2014 obtained from the base model, IRR_2_ indicates the corresponding IRR after additional adjustment for environmental factors, and IRR_1_−1 represents the excess risk of dengue in 2002 or 2014 versus the reference years^22^,^23^.

## Results

### Time-lagging effects on dengue incidence and mosquito index

Table 1 presents the time-lagging effects of meteorological and mosquito factors on monthly dengue incidence. In single factor analyses, all environmental parameters were associated with dengue risk, with minimum temperature having the greatest model fit (smallest QICu 1709.0). The best-fitting multivariate model, with the smallest QICu of 1536.8, was obtained from a complete GEE regression analysis. A higher minimum temperature rendered a constantly higher dengue risk at 1-month lag (β = 0.251, *P* = 0.018) and an interactively higher risk at 2- and 3-month lags (β = 0.080 for interaction, *P* = 0.001). The 1-month lagging effect of increased rainfall on dengue risk was observed to be negative (β = −0.194, *P* < 0.001), and the decreased risk was interactively reduced by the 2-month lagging effect of increased rainfall (β = 0.046 for interaction, *P* < 0.001).

Table 2 shows the time-lagging effects of minimum temperature and rainfall on dengue mosquito density index. Minimum temperature and precipitation were observed to have significantly positive 1- and 2-month lagging effects on BI value (β = 0.204 and 0.462, *P* = 0.001 and 0.029, respectively).

### Effects of the PGE on dengue incidence

**Figure 1:**
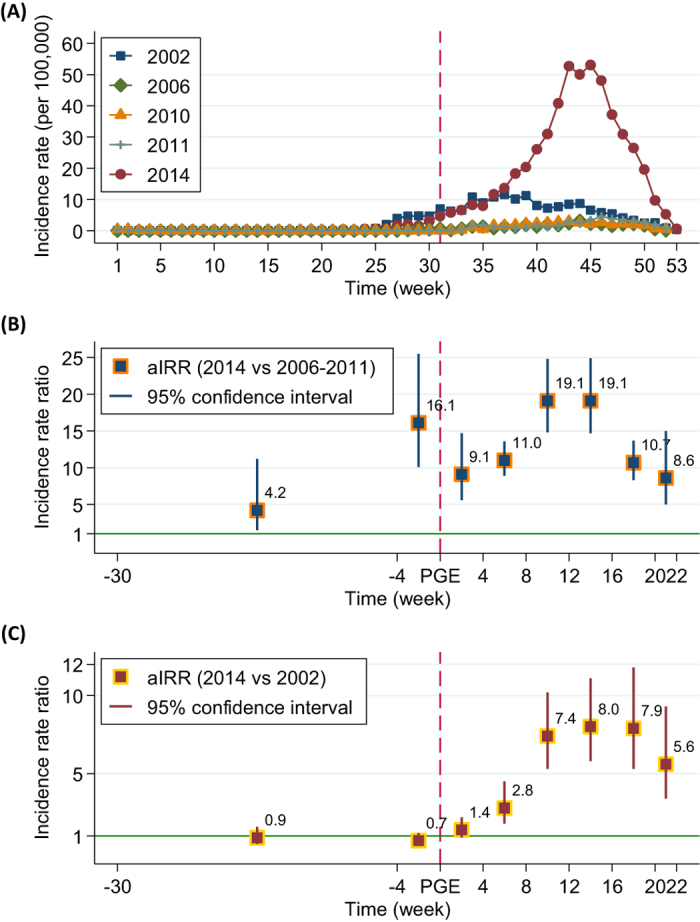
Distributions of weekly dengue incidences (100,000^−1^) in large-scale (2002 and 2014) and moderate-scale (2006, 2010 and 2011) outbreak years (**A**), and adjusted incidence rate ratios (aIRR) of dengue for the periods before and after petrochemical gas explosion (PGE) occurred at the 31th week (shown in a red dashed line), compared 2014 with 2006 + 2010 + 2011 (**B**) and with 2002 (**C**), Kaohsiung, Taiwan. **Note:** aIRRs were adjusted for maximum, minimum, average temperatures, relative humidity, rainfall and the percentage of Breteau index level >2 in a month.

Figure 1 illustrates the distributions of weekly dengue incidences in the large- and moderate-scale outbreak years and presents the effects of PGE on dengue incidence. The dengue risk in 2014 was similar to that in 2002 (aIRR = 0.7‒0.9, *P* > 0.05) but not to that in other years (aIRR = 4.2‒16.1, *P* < 0.05) before the PGE. Accordingly, the distribution of weekly dengue incidences in 2002 was considered a reference for evaluating the possible effect of PGE. Compared with dengue incidences in the corresponding period in 2002, dengue risk was nonsignificant for the first 4 weeks after PGE (aIRR = 1.4, *P* > 0.05). However, a remarkably higher risk was observed from week 5 after the PGE (aIRR = 2.8 for weeks 5‒8, *P* < 0.05), with elevated risks covering all periods after the PGE (aIRR = 2.8‒8.0 for weeks 5‒22), even after controlling for all environmental parameters.

**Figure 2:**
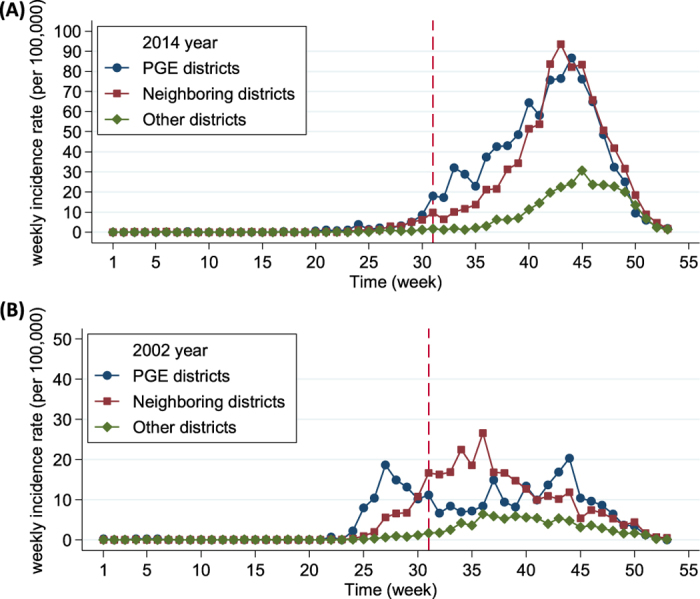
Distributions of weekly dengue incidences (100,000^−1^) in petrochemical gas explosion (PGE) affected districts (Qianzhen and Lingya), PGE neighboring districts (Sanmin, Xiaogang and Fengshan) and the other administrative districts (33 districts in total) in 2014 (**A**) and in 2002 (**B**), Kaohsiung, Taiwan. **Note:** The PGE event occurred at the 31th week (July 30) in 2014, as shown in a red dashed line.

Figure 2 shows the distribution of weekly dengue incidences in the PGE-affected districts, their neighboring districts, and the other administrative Kaohsiung districts in 2002 and 2014. After the PGE in 2014, an increasing trend of dengue incidences was observed in the PGE-affected districts earlier than in their neighboring districts (Fig. 2A). The incidence of dengue in the other administrative districts also increased, but later and with a milder trend. In the corresponding periods and districts, no compatible increases in trends of dengue incidences were observed in 2002 (Fig. 2B).

### Cross-time relationship of dengue incidence between PGE affected and neighboring districts

Table 3 presents the cross-time associations of weekly dengue incidences between different districts. The cross-correlation of the dengue incidences in the PGE-affected districts with those in the PGE-neighboring districts during 1‒4 weeks after the PGE was 0.672‒0.956 (*P* < 0.05), with the greatest correlation observed at the first week after the PGE. The highest correlation was observed between the dengue incidences of the PGE and other administrative districts at the third week after the PGE (*r*_s_ = 0.963, *P* < 0.001). By contrast, no significant cross-time associations between dengue incidences were identified in 2002.

### Excess risk of dengue explained by environmental factors

The assessment of the excess risk of dengue explained by significant time-lagging effects of environmental factors (variables in the best-fitting model) and the PGE in 2002 and 2014 is presented in Table 4. After adjusting for the 1‒3-month lagging effects of minimum temperature and the 1- and 2-month lagging effects of rainfall (both including interaction), the aIRR of dengue decreased to 7.9 in 2002 and 19.7 in 2014; these lagging effects separately explained 32.4% and 44.1% of excess risk of dengue incidence for 2002 and 2014, respectively. The combined effect of the 2 meteorological parameters and the PGE accounted for 75.1% of the excess risk of dengue in 2014. The excess risk patterns for dengue observed during the hot incident period (June to December, Supplementary Information Figure 2) were similar to those observed for the entire year, with a slightly higher proportion of excess risk in 2014 explained through minimum temperature, rainfall, and PGE (76.5%).

### Differences in minimum temperature and precipitation across study years

**Figure 3:**
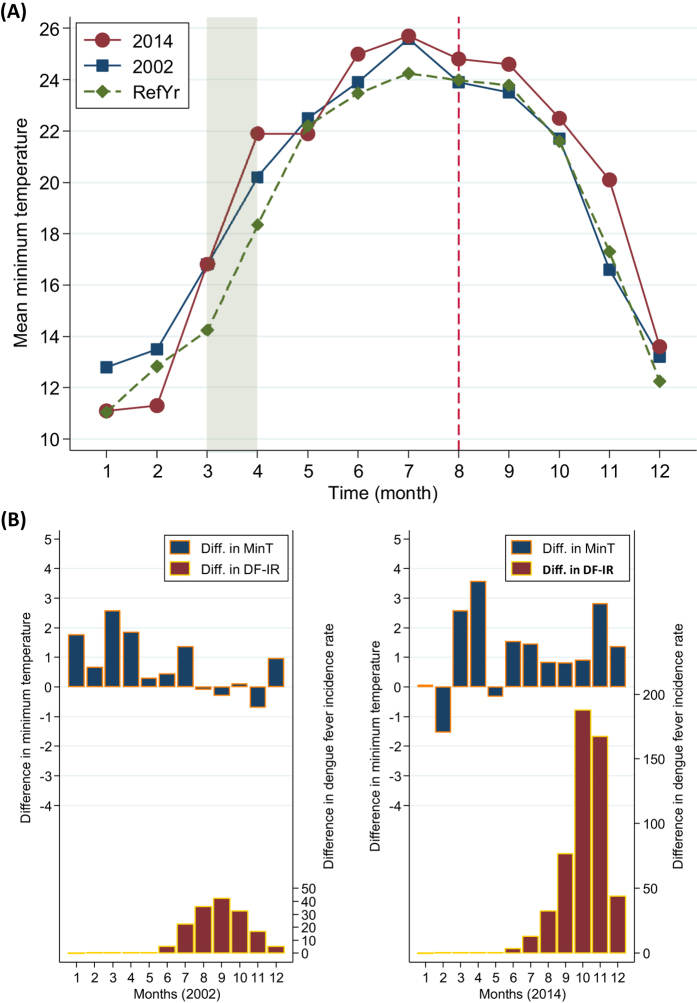
Distributions of monthly average minimum temperature (MinT) (°C) in 2002, 2014 and reference years (RefYr, all years except 2002 and 2014) (**A**); and MinT differences (Diff.) and dengue fever incidence rates (DF-IR, 100,000^−1^), compared 2002 and 2014 with reference years (**B**), Kaohsiung, Taiwan. **Note:** The notable difference in MinT starting from March to April was consistently found for 2002 and 2014 (shadowed area), a period of 2 to 3 months before the hot period of dengue occurrence (June to December). The PGE event occurred at July 30 in 2014 (shown in a red dashed line).

Figure 3 illustrates the distributions and differences in monthly average minimum temperatures for study years. Compared with those in the reference years, notable discrepancies were consistently observed in the minimum temperatures from March to April in 2002 and 2014, a period of 2 to 3 months before the hot period of dengue occurred. Data on monthly average precipitation revealed that lower rainfall (ie, higher aridity) was associated with higher dengue incidences, particularly from June to October, and the association was more apparent in 2002 than in 2014 (Supplementary Information Figure 3).

## Discussion

This study reports the time-lagging effects of minimum temperature and rainfall on dengue incidence, presents findings regarding the time required for PGE to affect dengue incidence, and the combined effect of meteorological parameters after a time lag and PGE on dengue incidence in Kaohsiung.

The time-lagging effects of climate variables on dengue occurrence have been investigated in numerous areas to which dengue is endemic^24^,^25^,^26^,^27^. In Bangladesh, temperature and humidity was associated with dengue incidence, with the greatest effects at a 2-month lag (2000–2010)^24^, and in Indonesia, a 1-month lag in temperature and a 2-month lag in precipitation accounted for the highest variability in the association between weather parameters and dengue incidence (1992–2001)^25^. The greatest dengue effects from average temperature in Puerto Rico (1988−1992) and minimum temperature in the Caribbean islands of Barbados (1995−2000) were also observed at a 3-month lag^26^,^27^. In the current study, assessing long-term dengue data (2000–2014) by using a completed environmental parameter-included model revealed that a 2- and 3-month lag in minimum temperature and a 1- and 2-month lag in rainfall have interactively increased and decreased effects on dengue incidence, respectively. Furthermore, minimum temperature and precipitation had significant lagging effects on mosquito density index. The varying degrees of association and interaction between weather, mosquito, and host characteristics in different regions may partly explain the regional differences in the lagging effects of environmental factors; however, our findings suggest that increased minimum temperatures 2 and 3 months before dengue occurrence are interactively correlated with an early occurrence of an environment favoring the growth, survival, and dispersion of mosquito vectors, and that drier conditions (ie, lower rainfall) 1 and 2 months before dengue occurrence are interactively correlated with near surroundings that increase the risk of dengue transmission in tropical Kaohsiung.

PGEs themselves do not cause dengue infections; nonetheless, the mosquito‒human dynamics affected by such events may directly or indirectly promote disease transmission. The potential mechanisms of transmission in the current case are as follows: (1) the gutters were destroyed in the blast area, and dengue vector mosquitos living beneath the street flew out to damaged houses, potholes, and other habitats; (2) the disaster zone was blocked, thus epidemic prevention programs for dengue mosquito-breeding source clearance and chemical mosquito control were interrupted in the first week after the PGE; (3) because of several days of rainfall after the occurrence of the PGE and several weeks of hot weather thereafter, mosquito-breeding sites in the number of open spaces increased; and (4) the mobility of infected (virus-carrying) people and dengue vector mosquitoes living in and around the disaster zone increased, thus the dengue viruses spread to neighboring districts.

Compared with the epidemic pattern of dengue incidence in 2002, a significantly higher dengue risk was noted from week 5 after the PGE in 2014, providing empirical information for disease control after similar incidents influencing mosquito‒human dynamics. This study also revealed that dengue incidences in all PGE-affected districts, their neighboring districts, and other administrative Kaohsiung districts increased significantly after the PGE, with the highest cross-time correlation of dengue incidences in the PGE-affected districts with their neighboring and other districts observed at 1 and 3 weeks after the PGE, even when no comparable patterns were noted in 2002. Data on dengue vector mosquito monitoring has shown that the number of *Li*, the smallest administrative division in Taiwanese cities, with BI >2 in the nonexplosion zones of the PGE-affected districts increased from 5 at week 31 to 15 at week 35 (accurate survey data for accident zones were unavailable because these zones were blocked)^8^. These findings support the argument that PGE-affected environments may be correlated with the spread of virus-carrying mosquitoes and contribute to dengue infection.

Entomological studies have demonstrated that the larval survival, adult growth rate, reproduction length, and feeding behavior of *Aedes aegypti* are temperature-dependent^9^,^28^,^29^. The curve of temperature-related mortality rates of *Aedes* mosquitoes in aquatic and adult phases was observed to be U-shaped, with optimal survival at 15 °C−35 °C and 15 °C−30 °C, respectively^28^. The temperature range of 20 °C−30 °C was associated with the highest survival rate of 88%−93% from first-stage larvae to adulthood^29^, and <15 °C and >36 °C have a limited or ceased feeding activity^9^. This study revealed that the 2- and 3-month lagging interplay effect and 1-month lagging effect from minimum temperature accounted for 18.0% and 42.9% of excess dengue risk in 2002 and 2014, respectively. In Kaohsiung, March and April denoted the period of 2 and 3 months before of the onset of the dengue epidemic in June. Our study revealed that the mean minimum temperature in 2002 and 2014 was approximately 2 °C−4 °C higher than that in the reference years during March and April (16.8 °C and 20.2 °C−21.9 °C in 2002 and 2014, respectively, and 14.2 °C and 18.3 °C in the reference years, Fig. 3). The mean minimum temperature in March of 2002 and 2014 (both 16.8 °C) was within the optimal temperature range for survival (15 °C−35 °C) and that in April of 2002 and 2014 (20.2 °C−21.9 °C) was within the range with a highest survival rate (20 °C−30 °C); however, no comparable temperature conditions were observed in the reference years for either. These findings emphasize that an increased minimum temperature 2–3 months before the onset of a dengue epidemic may be a crucial determinant associated with dengue outbreak in Kaohsiung. This phenomenon also reflects the impact of global warming: the temperature at night increases faster than it does at daytime^30^.

Although rainfall offers a vital habitat for the aquatic stages of the mosquito lifecycle, drier conditions may increase the duration of mosquito-feeding activity, thus increasing the vector infectious period^31^. Our study revealed that higher rainfall has a 2-month lagging effect on the higher dengue mosquito density index, whereas lower rainfall renders 1- and 2-month lagging effects on higher risks of dengue occurrence. These findings indicate the complex mechanisms through which meteorological parameters affect dengue transmission.

A recent environmental investigation assessing laboratory, field, and statistical data concluded that the interaction between dengue ecology, virus development, and host species constitutes a complex dynamic that affects dengue transmission^9^. This study revealed that the combined effect of increased minimum temperature, decreased rainfall, and the PGE explained 75.1% of the excess dengue risk for all of 2014 and 76.5% for June to December of that year. Because the PGE occurred in the hot epidemic period of DF (June to December), its impact might have extended the effect of minimum temperature and rainfall on dengue incidences: 98.2% (75.1%/76.5%) of excess dengue risk of the entire year was associated with the 3 factors. The PGE itself does not induce dengue epidemics; rather, the affected mosquito‒human dynamics might have interacted with temperature and rainfall parameters to influence dengue infection dispersion.

This study has several strengths. First, it provides the first and most comprehensive dengue data over a recent long-term period (2000–2014) and evaluates the combined effects of meteorological/mosquito factors and a PGE on dengue incidence in a metropolitan city at high risk of dengue epidemics. Second, the complete time-lagging interactive effects of environmental parameters were considered and analyzed over relatively short periods (ie, weeks and months). However, this investigation has some limitations. First, this was an ecological association evaluation and could not provide a causal inference at individual levels. Second, data on nonmeteorological covariates, such as herd immunity and vector control programs, were unavailable; thus, the associated confounding effects could not be controlled. Finally, the findings are only applicable to areas with comparable weather and socioecological backgrounds; however, the assessment approaches for dengue incidence can be applied to area-specific data to determine local determinants.

In conclusion, time-lagging interplay effects from minimum temperature and rainfall may be respectively associated with early and near environments facilitating dengue transmission. New types of events, such as PGEs, which differ from weather abnormalities but are associated with alterations in mosquito‒human dynamics, should be carefully considered during dengue prevention and control, because they can interact with meteorological parameters and affect the spread of dengue infection.

## Additional Information

**How to cite this article**: Chang, K. *et al*. Time-Lagging Interplay Effect and Excess Risk of Meteorological/Mosquito Parameters and Petrochemical Gas Explosion on Dengue Incidence. *Sci. Rep*. **6**, 35028; doi: 10.1038/srep35028 (2016).

## Supplementary Material

Supplementary Information

## Figures and Tables

**Table 1:** Time-lagging effects (β)^a^ of meteorological and mosquito factors on monthly dengue incidence, Kaohsiung, Taiwan, 2000–2014.

Factors	Temperature (°C)													
	Minimum		Maximum		Average		Range		Relative humidity (%)		Rainfall (100 mm)		Breteau index value > 2 (%)	
	β	*P*	β	*P*	β	*P*	β	*P*	β	*P*	β	*P*	β	*P*
Single factor model^b^														
Main effect														
1-month lag	0.142	0.011	1.052	0.059	0.254	0.015	−0.201	0.051	−0.067	0.074	0.078	0.369	−0.017	0.435
2-month lag	0.109	0.079	1.024	0.008	0.293	0.256	−0.255	0.410	0.267	<0.001	0.219	0.001	0.030	0.340
3-month lag	0.300	<0.001	0.384	0.058	0.391	0.004	−0.554	0.002	0.301	<0.001	0.197	<0.001	−0.002	0.931
Interaction effect														
1-month lag x 2-month lag			−0.395	0.017	−0.112	0.003					−0.028	0.027	−0.002	0.027
1-month lag x 3-month lag											−0.074	0.037		
2-month lag x 3-month lag	0.064	0.004			0.067	0.006			−0.028	0.005				
Model QICu:	1709.0		2083.9		1985.1		2212.0		2788.1		2708.8		2560.0	
Multiple factor model^c^														
Main effect														
1-month lag	**0.251**	0.018									−0.194	<0.001		
2-month lag	0.151	0.192									−0.069	0.233		
3-month lag	0.289	0.002												
Interaction effect														
1-month lag x 2-month lag											**0.046**	<0.001		
2-month lag x 3-month lag	**0.080**	0.001												
Model QICu:											**1536.8**	**(total)**^d^		

^a^GEE regression coefficient (β) denotes adjusted effect of covariates on dengue incidence and exp(β) expresses adjusted incidence rate ratio.

^b^Single environmental factor with all time-lagging effects (including interactions) were evaluated simultaneously in a regression model.

^c^Multiple environmental factors with all time-lagging effects (including interactions) were evaluated simultaneously in a regression model. Only the best-fitting regression model was shown in the table.

^d^The model QICu for the best-fitting multiple factor regression model.

**Table 2:** Time-lagging effects (β)^a^ of minimum temperature and rainfall dengue vector mosquito density index^b^, Kaohsiung, Taiwan, 1–6 months, 2000–2014.

Factors	β	(95% CI)	*P* value
Minimum temperature (°C)			
Concurrent	0.046	(−0.045, 0.137)	0.317
1-month lag	**0.204**	(0.081, 0.328)	0.001
2-month lag	−0.080	(−0.166, 0.006)	0.069
3-month lag	0.065	(−0.001, 0.131)	0.052
Rainfall (100 mm)			
Concurrent	0.007	(−0.048, 0.063)	0.793
1-month lag	0.047	(−0.110, 0.204)	0.556
2-month lag	**0.462**	(0.047, 0.878)	0.029
3-month lag	−0.727	(−1.821, 0.368)	0.193

^a^GEE regression coefficient (β) was adjusted for all time-lagging effects for minimum temperature and rainfall in the Table.

^b^The proportion of the days with a Breteau index level >2 in a month was used as dengue vector mosquito density index.

**Table 3:** Cross-correlations (*r*_s_)^a^ between weekly dengue incidences in petrochemical gas explosion-affected districts and subsequent weekly dengue incidences in the other districts, Kaohsiung, Taiwan, 2002 and 2014.

Time status	2014		2002	
	PGE-AD and PGE-ND	PGE-AD and Others	PGE-AD and PGE-ND	PGE-AD and Others
Concurrent	0.939***	0.711*	0.419	0.670*
Time after the PGE				
1-week	0.956***	0.866***	0.272	0.499
2-week	0.916***	0.959***	0.201	0.286
3-week	0.822***	0.963***	0.102	0.207
4-week	0.672*	0.894***	−0.076	−0.111
5-week	0.513	0.757**	−0.274	−0.310
6-week	0.375	0.600	−0.406	−0.383
7-week	0.226	0.404	−0.446	−0.615

PGE: petrochemical gas explosion; PGE-AD: PGE-affected districts; PGE-ND: PGE-neighboring districts; Others: the other administrative districts.

^a^Spearman’s rank correlation coefficients (*r*_s_) with Bonferroni-adjusted *P* values were used to evaluate time cross-correlations; Only incidence data after the PGE was assessed (a total of 23 weeks after 31 July, *P < 0.05; **P < 0.005 and ***P < 0.001).

**Table 4:** Incidence rate ratios of dengue fever and excess risks in 2002 and 2014 explained by time-lagging effect of environmental factors and petrochemical gas explosion event in diverse time periods, Kaohsiung, Taiwan.

Models	1–12 months					6–12 months				
	RefYr	2002		2014		RefYr	2002		2014	
	Ref.	IRR	ERE^b^	IRR	ERE^b^	Ref.	IRR	ERE^b^	IRR	ERE^b^
**Base model (BM)**^a^	**1.0**	11.2*		34.5*		**1.0**	11.5*		35.3*	
Model 1: BM + MinT (*i*-month lag, positive effect)										
MinT: 1-m lag + 2-m lag + 3-m lag + 2-m lag x 3-m lag	1.0	9.4*	18.0%	20.1*	42.9%	1.0	9.6*	18.0%	20.5*	43.1%
Model 2: Model 1 + Rainfall (*i*-month lag, negative effect)										
Rainfall: 1-m lag + 2-m lag + 1-m lag x 2-m lag	1.0	7.9*	32.4%	19.7*	44.1%	1.0	7.9*	34.1%	18.9*	47.7%
Model 3: Model 2 + PGE										
PGE: occurred on July 31, 2014	1.0	7.8*	—	9.3*	75.1%	1.0	8.0*	—	9.1*	76.5%

**Note**: *P < 0.05; IRR: incidence rate ratio; MinT: minimum temperature; PGE: petrochemical gas explosion; RefYr: reference years (the period during 2000 to 2014, but beyond 2002 and 2014; a total of 13 years).

^a^Base model measured the IRRs of 2002 and 2014 as compared to the reference years.

^b^Excess risks explained (ERE) by covariates additionally added to the study model.
