# Supplementary material for: Time-Lagging Interplay Effect and Excess Risk of Meteorological/Mosquito Parameters and Petrochemical Gas Explosion on Dengue Incidence
Source: Sci Rep. 2016 Oct 13;6:35028. doi: 10.1038/srep35028 (PMC5062066; doi:10.1038/srep35028)
Supplement: Supplementary Information [file srep35028-s1.pdf]

Time-Lagging Interplay Effect and Excess Risk of Meteorological/Mosquito Parameters and Petrochemical Gas Explosion on Dengue Incidence

Ko Chang[#1,2], Chaur-Dong Chen[#3], Chien-Ming Shih[4,5], Tzu-Chi Lee[6], Ming-Tsang Wu[5-7], Deng-Chyang Wu[8], Yen-Hsu Chen[1], Chih-Hsing Hung[4,9], Meng-Chieh Wu[2], Chun-Chi Huang[6,10], Chien-Hung Lee[5,6*], Chi-Kung Ho[3,6*]

1. Division of Infectious Diseases, Department of Internal Medicine, Kaohsiung Medical University Hospital, College of Medicine, Kaohsiung Medical University, Kaohsiung, Taiwan
2. Department of Internal Medicine, Kaohsiung Municipal Hsiao-Kang Hospital, Kaohsiung Medical University, Kaohsiung, Taiwan
3. Bureau of Public Health, Kaohsiung City Government, Kaohsiung, Taiwan
4. Graduate Institute of Medicine, College of Medicine, Kaohsiung Medical University, Kaohsiung, Taiwan
5. Research Center for Environmental Medicine, Kaohsiung Medical University, Kaohsiung, Taiwan
6. Department of Public Health, College of Health Science, Kaohsiung Medical University, Kaohsiung, Taiwan
7. Department of Community Medicine, Kaohsiung Medical University Hospital, Kaohsiung Medical University, Kaohsiung, Taiwan
8. Department of Internal Medicine, Kaohsiung Municipal Ta-Tung Hospital, Kaohsiung Medical University, Kaohsiung, Taiwan
9. Department of Pediatrics, Kaohsiung Municipal Hsiao-Kang Hospital, Kaohsiung Medical University, Kaohsiung, Taiwan
10. Department of Laboratory Medicine, Kaohsiung Municipal Hsiao-Kang Hospital, Kaohsiung, Taiwan

Supplementary Information Table 1. Distributions of meteorological parameters and mosquito index, Kaohsiung, Taiwan, 2000-2014

Lag time	Temperature (°C)								Relative humidity (%)		Rainfall (mm)		Breteau index level>2 (%)	
	Minimum		Maximum		Average		Range							
	Mean	SD	Mean	SD	Mean	SD	Mean	SD	Mean	SD	Mean	SD	Mean	SD
1-month lag	18.9	5.1	32.3	2.3	25.4	3.5	13.3	3.2	75.1	4.3	163.6	248.9	24.7	22.9
2-month lag	18.9	5.1	32.3	2.3	25.4	3.5	13.3	3.3	75.1	4.3	164.5	249.3	24.8	22.9
3-month lag	18.9	5.1	32.3	2.3	25.4	3.5	13.4	3.3	75.1	4.3	165.4	249.7	25.0	22.9

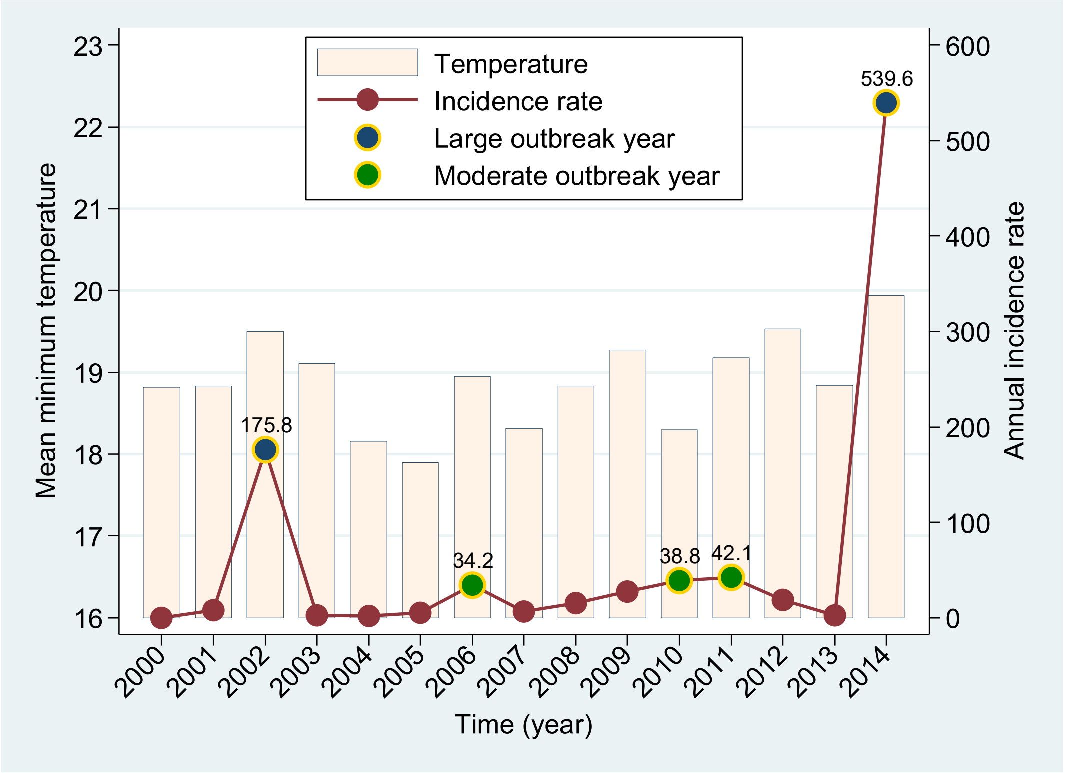

Supplementary Information Figure 1. Annual dengue incidence rates ($100{,}000^{-1}$) and average minimum temperatures (°C) in Kaohsiung, Taiwan, 2000-2014 (Spearman's rank correlation (r_s) between annual dengue incidences and annual average minimum temperatures is 0.611, $P = 0.016$; 2002 and 2014 are large-scale outbreak years, and 2006, 2010 and 2011 are moderate-scale outbreak years, all have dengue incidence rates >30 per 100,000 person-years).

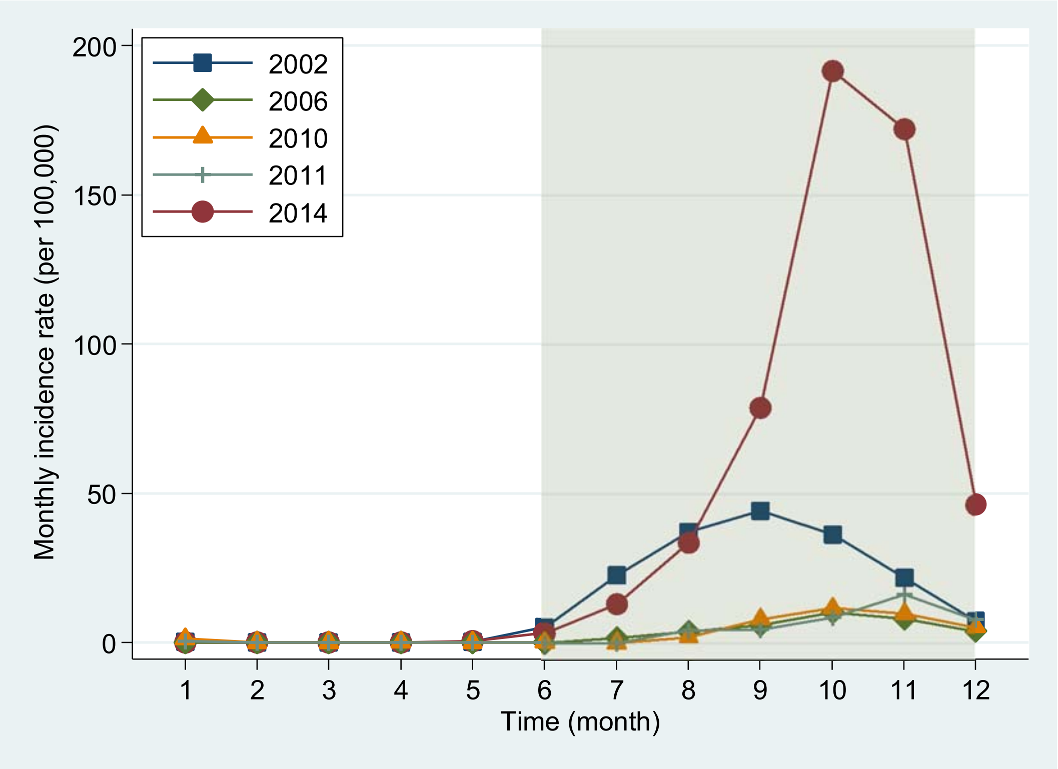

Supplementary Information Figure 2. Monthly dengue incidences (100,000-1) for large-scale outbreak years (2002 and 2014) and moderate-scale outbreak years (2006, 2010 and 2011), with a shadow for the hot incident period (6–12 months) in Kaohsiung, Taiwan.

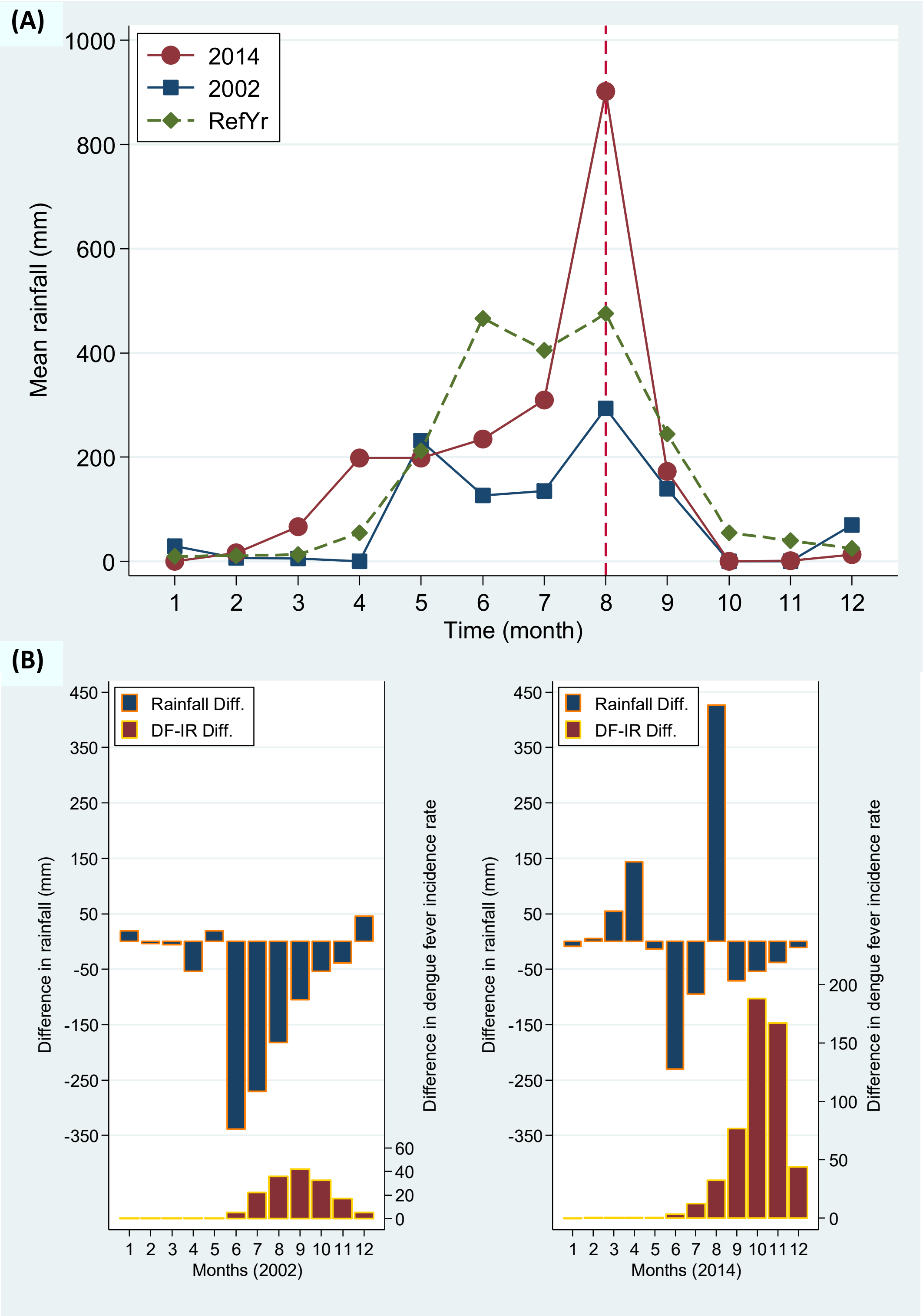

Supplementary Information Figure 3. Distributions of monthly average rainfall (mm) in 2002, 2014 and reference years (RefYr, all years except 2002 and 2014) (**Fig-A**); and rainfall differences (Diff.) and dengue fever incidence rate (DF-IR, 100,000^{-1}), compared 2002 and 2014 with reference years (**Fig-B**), Kaohsiung, Taiwan. The PGE event occurred at July 30 in 2014 (shown in a red dashed line).
